# Single cell classification of macrophage subtypes by label-free cell signatures and machine learning

**DOI:** 10.1098/rsos.220270

**Published:** 2022-09-28

**Authors:** David Dannhauser, Domenico Rossi, Vincenza De Gregorio, Paolo Antonio Netti, Giuseppe Terrazzano, Filippo Causa

**Affiliations:** ^1^ Interdisciplinary Research Centre on Biomaterials (CRIB) and Dipartimento di Ingegneria Chimica, dei Materiali e della Produzione Industriale, Università degli Studi di Napoli ‘Federico II’, Piazzale Tecchio 80, Naples 80125, Italy; ^2^ Center for Advanced Biomaterials for Healthcare@CRIB, Istituto Italiano di Tecnologia, Largo Barsanti e Matteucci 53, Naples 80125, Italy; ^3^ Dipartimento di Biologia, Università degli Studi di Napoli ‘Federico II’, Complesso Universitario di Monte S Angelo, Naples, Italy; ^4^ Dipartimento di Scienze (DiS), Università della Basilicata, Via dell'Ateneo Lucano 10, Potenza 85100, Italy

**Keywords:** single-cell, label-free, machine learning, optical signature, macrophages

## Abstract

Pro-inflammatory (M1) and anti-inflammatory (M2) macrophage phenotypes play a fundamental role in the immune response. The interplay and consequently the classification between these two functional subtypes is significant for many therapeutic applications. Albeit, a fast classification of macrophage phenotypes is challenging. For instance, image-based classification systems need cell staining and coloration, which is usually time- and cost-consuming, such as multiple cell surface markers, transcription factors and cytokine profiles are needed. A simple alternative would be to identify such cell types by using single-cell, label-free and high throughput light scattering pattern analyses combined with a straightforward machine learning-based classification. Here, we compared different machine learning algorithms to classify distinct macrophage phenotypes based on their optical signature obtained from an ad hoc developed wide-angle static light scattering apparatus. As the main result, we were able to identify unpolarized macrophages from M1- and M2-polarized phenotypes and distinguished them from naive monocytes with an average accuracy above 85%. Therefore, we suggest that optical single-cell signatures within a lab-on-a-chip approach along with machine learning could be used as a fast, affordable, non-invasive macrophage phenotyping tool to supersede resource-intensive cell labelling.

## 1. Introduction

Macrophages play essential roles in immune responses. These cells play a primary role during the innate immune response and, with phagocytosis, contribute to the elimination of microbes, foreign molecules and cellular debris [1–3]. In the adaptive and antigen-specific phases of the immune response, macrophages actively play the role of antigen-presenting cells (captured and processed during phagocytosis) to T lymphocytes. Furthermore, macrophages are involved in numerous inflammatory processes such as cancer, atherosclerosis and diabetes [4,5]. Macrophages represent the resident cells in peripheral tissue derived from blood circulating monocytes that can extravasate from the blood stream and migrate toward inflammatory and/or infection sites recruited by chemokine gradients [6]. During the migration in peripheral tissues, monocytes transform into mature macrophages, presenting significantly different cell properties, such as large cell volume, an activated nucleus and cytoplasm composition (numerous vesicles and mitochondria). Yet their characteristics are not uniform through the whole macrophage population, mainly depending on the environment they migrate toward and chemokines they encounter. Macrophages can be classified from the polarization process [7–9], the chemokines for their stimulation [10] and the involved cell pathways [11], macrophages into two polarization paths called M1 and M2 phenotypes. M1 are classically activated macrophages, inducing pro-inflammatory peripheral tissue milieu, by cytokine secretion, and exhibiting potent antimicrobial properties through production of nitric oxide and radical oxygen intermediates, fostering lymphocytes T-helper responses. In contrast, M2 are alternatively activated macrophages, which act in the opposite way (pro-regenerative), inducing immune downregulation and tissue remodelling such as wound healing and fibrosis [12,13]. Moreover, M2 produce cytokines with antiatherogenic and profibrotic properties, which promote plaque stability.

Macrophages play a crucial role in either maintaining metabolic homeostasis and, if mis-regulated, in the progression of abundance of inflammatory-mediated diseases by shifting the M1 versus M2 balance toward either M1 or M2 phenotypes [5,12,13]. Therefore, an imbalance between M1 and M2 phenotype equilibrium can be strictly correlated with a number of pathologic conditions, such as tissue fibrosis, which can lead to the loss of organ functions [14–16] as well as inflammations [17], tuberculosis [18], severe obesities [19], diabetes mellitus [20], rheumatoid arthritis [21], complication during pregnancies [22] or cancers [23]. Also, in wound healing during the acute phase, M1 macrophages dominate, while proper wound regeneration processes shift the balance toward M2-like macrophage phenotypes. To date, many markers exist to characterize and distinguish M1 from M2 phenotypes by histochemical reactions, such as cluster differentiation molecules (CD68 for M1 and CD163 for M2), chromatin condensation or mRNA expressions (TNF-*α* for M1 and IL-10 for M2 recognition) [24–28].

A macrophage phenotype polarization type recognition can be achieved with one—or more—of the previously mentioned methods, but all of them suffer from significant technological drawbacks. In general, pre-processing of the biological sample is mandatory, and such procedures are usually time consuming, cost-effective and, more important, they modify internal and external cell structures of the investigated cells. Today, to the best of our knowledge, there are few methods to distinguish M1 from M2 phenotype that prevent the mentioned technological drawbacks. For instance, Bertani *et al.* [29] use a novel technique based on reflectance confocal microscopy and multivariate analysis. However, a label-free approach to investigate possible structural differences between M1 versus M2 phenotype and classify them is still missing.

**Figure 1.**
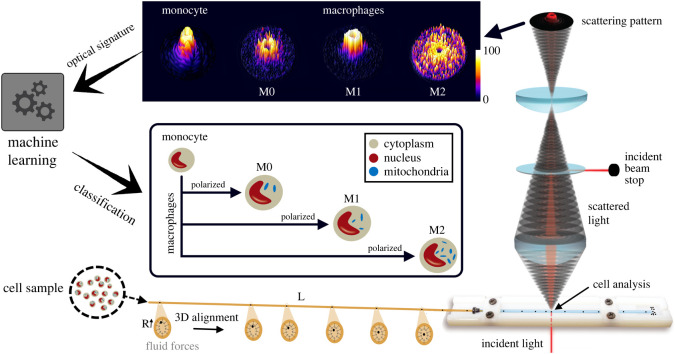
Experimental set-up of label-free approach to classify polarized macrophage subtypes based on in-flow optical signature investigation. Fluid forces three-dimensionally align cells from a cell sample to the centreline of a microfluidic device (from the left to the right), where a collimated laser beam (incident light) interacts with passing individual cells. The light interaction reveals significantly different scattering patterns (optical signature) for each macrophage phenotype as well as monocytes, which a camera-based readout system record. The obtained data are processed and classified with machine learning to obtain a label-free macrophage phenotype classification. The inset indicates the classified morphological differences between the different macrophage phenotypes correlated to the distinct illustrative optical signatures shown above.

In this manuscript, we present a straightforward method to classify polarized macrophage subtypes by their morphometric point of view using a non-destructive light scattering approach [30] combined with machine learning-based single cell classification (figure 1) [31]. Compared with standard flow cytometric approaches—which are known to have high instrumentation and service costs—the presented method is very simple and cost-effective, permitting a classification of cell subtypes without large numbers of cells and resource-intensive labelling. More importantly, measurements are realized using a lab-on-a-chip approach permitting the measurement of living cells in suspension, which furthermore are collectable and re-usable for other diagnostic investigations or therapeutic approaches. We have already investigated such optical signature of other human cell types and subtypes [32–36]. Here, we focused on intracellular differences of macrophages regarding dimensional and optical point of view. It is known that M2 present a greater mitochondrial density compared with M1, which can lead to more pronounced side scattering profiles of single cells [37]. Therefore, optical cell signatures obtained from our wide static light scattering approach can significantly improve macrophage phenotype investigations by providing a morphological characterization of cells in suspension. Such fingerprint of a given phenotype is the basis of the present approach to distinguish among monocytes and different macrophage phenotypes.

## 2. Results and discussion

**Figure 2.**
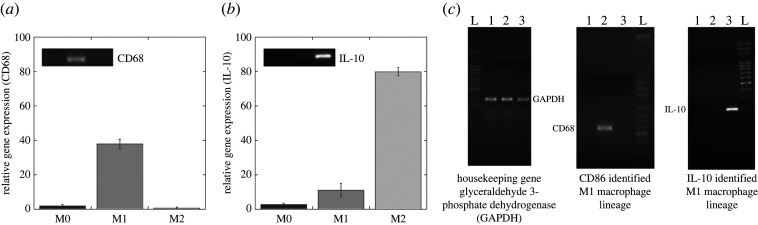
RT-PCR analysis of cluster differentiation genes CD68 (*a*), IL-10 (*b*) and results from the agarose gel stained with ethidium bromide (*c*). The graphs evidence the relative gene expression normalized by the housekeeping gene GAPDH. The insets highlight the specific agarose gel result. Values in (*a*) and (*b*) represent the mean and the standard deviation (*n* = 3). L = DNA Ladder, 1, 2 and 3 = M0, M1 and M2 macrophage phenotypes, respectively. All cells were recovered from a healthy donor.

The different macrophage phenotypes (M0-unpolarized, M1- and M2-polarized) investigated in this work were first examined via molecular analysis using reverse transcription polymerase chain reaction (RT-PCR) [38] to confirm their polarization state. Data showed a relevant upregulation of CD68 gene expression in M1 samples (37.7 ± 2.79 arb. units) compared with M0 and M2. No relevant CD68 signal was found in M0 and M2 samples. Instead, M2 exhibited a significant upregulation of IL-10 (79.8 ± 2.45 arb. units) compared with M0 and M1, demonstrating the acquisition of M2-polarized macrophage phenotypes. A slight non-significant IL-10 expression (11.02 ± 3.9) was revealed in M1 samples (figure 2).

**Figure 3.**
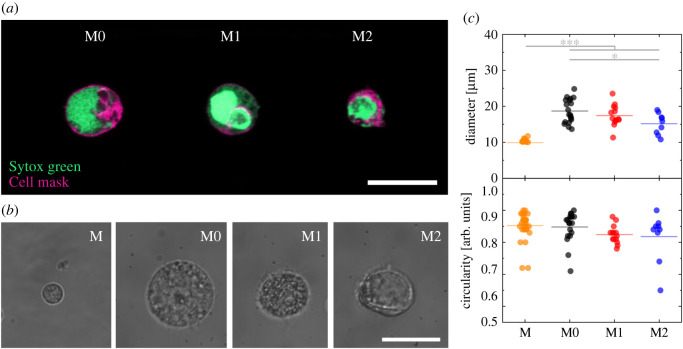
Fluorescence and bright-field investigation of monocytes and M0, M1 and M2 macrophage phenotypes recovered from a healthy donor. (*a*) Confocal investigations were obtained with a 63× objective using a 6.5× zoom factor. Cells of one donor are stained and coloured with Sytox Green to highlight cell nucleus and Cell Mask to indicate cell membrane structures. (*b*) Bright-field investigation of monocytes (M) and macrophage types (M0, M1 and M2 from left to right) directly before an in-flow experiment using a 100× magnification. (*c*) Diameters and circularity of bright-field investigations from one donor (*n* = 30, 19, 14 and 9 for M, M0, M1 and M2, respectively; *F* = 64.1 and 2.1 for diameter and circularity, respectively). The length of the scale bar is 20 µm. Statistical significances were determined by one-way ANOVA and Tukey's test (n.s. *p* > 0.05; *p < 0.05; ***p < 0.001).

To classify suspended macrophage phenotypes according to their morphological properties, we investigated their main cellular structures after fluorescence staining using a standard confocal microscope. In more detail, we used a green nucleic acid stain to highlight nuclear and chromatin content combined with a plasma membrane staining to indicate membrane structures. Our observations revealed an evident structural difference of cell cytoplasm contents between M1 versus M0 or M2 macrophage phenotypes, while M0 versus M2 macrophage phenotypes show similar cell staining results (figure 3*a*). Before each scattering experiment cells were observed at quiescent bright-field condition to investigate possible structural alterations (figure 3*b*) [38]. It is well known that macrophages present different sizes and shapes in tissue compared with suspension. Moreover, the cell detaching method can alter macrophages shape and properties. For instance, trypsin can down-modulate the surface CD163 level for M2 macrophages [39]. Therefore, we decided to use a cell scraper to minimize possible cell recovery issues after the detachment procedure. However, we observed in suspension a general round shape for all macrophage phenotypes (figure 3*c*), while characteristic cytosolic granules were observed. Cell observations result in a cell circularity ≥92% for all investigated macrophage phenotypes (figure 3*c*), which confirms a physiological cell shape after the detaching method and before in-flow scattering experiments. Furthermore, we observed a median monocyte diameter [38] with 9.89 ± 0.61 µm and macrophage diameter [38] variations from 18.67 ± 3.23 to 17.41 ± 2.85 µm and down to 15.15 ± 2.74 µm, for M0, M1 and M2, respectively.

**Figure 4.**
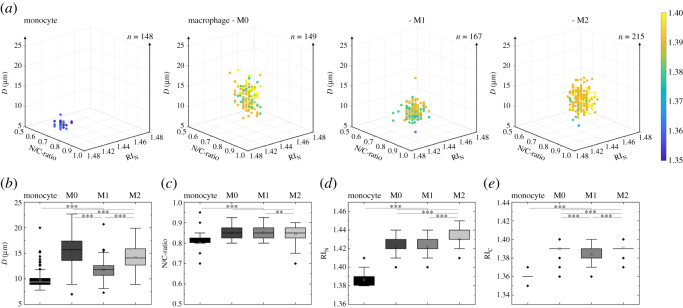
Three-dimensional scatter plots (*a*) and detailed biophysical cell property plots (*b*–*e*) of pooled monocytes and macrophage phenotypes data recovered from three healthy donors, with *n* = 148, 149, 167 and 215 for monocytes, M0, M1 and M2 phenotypes, respectively. *F* = 221, 108, 1083 and 1042 for D (*b*), N/C-ratio (*c*), RIN (*d*) and RIC (*e*), respectively. Statistical significances were determined by one-way ANOVA and Tukey's test (n.s. *p* > 0.05; **p < 0.01; ***p < 0.001).

After quiescent cell structure observations, we performed separate in-flow optical signatures analysis—using the microfluidic-based experimental set-up indicated in figure 1—to investigate in more detail morphological cell feature differences between monocytes, M0, M1 and M2 macrophage phenotypes. Figure 4*a* summarizes in illustrative three-dimensional scatter plots the biophysical properties [38] obtained from optical cell signatures (pooled data) and below detailed information of statistical relevant property differences between the different cell types. Not surprisingly, substantial property changes are visible in the overall cell dimension (figure 4*b*), from monocytes with 9.82 ± 1.59 µm to macrophage phenotypes, which range from approximately 11 to 16 µm. In more detail, M0 are the biggest cells with 15.66 ± 2.84 µm in dimension compared with 11.76 ± 1.72 µm and 14.25 ± 2.29 µm for M1 and M2 phenotypes, respectively. Such dimension differences are in good agreement with literature, where M0 and M2 cells are far bigger than M1 phenotypes [37]. Finally, nucleus over cytoplasm ratio (figure 4*c*) of the three macrophage phenotypes is found to be similar among each other with a ratio of 0.85, while being significantly different to their origin cell type with approximately 0.80. As next step, we investigated the optical response of the nucleus (*RI_N_*, figure 4*d*) between the different cell types. Hereby optical signatures revealed similar values for M0 and M1 phenotypes with a refractive index of approximately 1.42 compared with approximately 1.43 and approximately 1.39 for the M2 phenotype and monocytes, respectively. Finally, the optical response of the cell cytoplasm (*RI*_C_, figure 4*e*) was investigated from cell signatures, showing similar values for M0 and M2 phenotypes with approximately 1.39 compared with approximately 1.38 and approximately 1.36 for the M1 phenotype and monocytes, respectively.

Our outcome confirms morphometric cell property changes during cell polarization. In fact, it is well known in literature that the macrophage cytoplasm consists of lysosomes, mitochondria and granules, which composition can change for different phenotypes. From a scattering point of view, the contribution of inner cell structures depends on their dimension, number, position and composition (e.g. cytokine type and concentration). In fact, the optical signature of M2 macrophages illustrates significant scattering differences compared with other phenotypes (figures 1 and 4). For instance, a significantly higher *RI_N_* value is detected compared with M0 and M1 phenotypes, which could simply imply a more active cell state or other cell content which is recognized as nucleus. Note that our single-cell scattering approach simplifies a cell as core–shell construct. However, it has been demonstrated that a change in optical density of nucleus can be related to an active state of chromatin (active state of gene transcription). Regarding this, Rostam *et al.* observed that the fluorescence intensity of nuclear staining is significantly low for M2 cells compared with M1, clearly showing a more intense activity of M2, which may be related to the different action of transcriptional factors on M1 and M2 polarized macrophages [40,41]. Interestingly, the work of Halaney *et al.*, reports a detailed analysis of light scattering distribution of M1 and M2 macrophage phenotypes [37]. The authors found that M1 and M2 present a significantly different amount of scattering intensity at side angles between 2 and 3°, which is in good agreement with our findings (see optical signature distributions in figure 1) for M2 compared with M0 or M1 phenotypes. Compared with Halaney *et al.*, a significantly wider scattering angle range (2–30°) can be observed with our measurement technique, at single cell level resolution [37]. In fact, such additional information allows a more detailed macrophage subtype measurement, with a higher cell throughput rate, thanks to our microfluidic-based measurement concept. From a structural point of view, such behaviour seems to be related to small inner structure differences such as nuclei, lysosomes or/and mitochondria. In fact, mitochondria are known to add a significant contribution at side scattering angles, due to their dimension, optical density and structural location in the cell cytoplasm [37,42].

**Figure 5.**
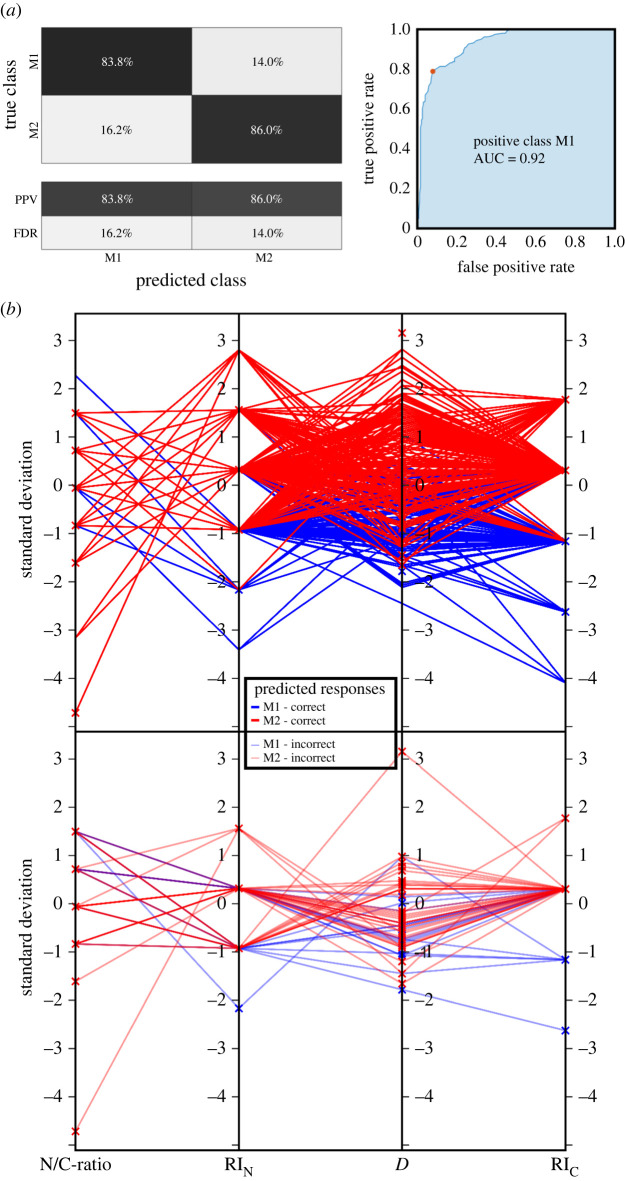
ML classification of M1 versus M2 polarized macrophage phenotypes from scattering outcome recovered from three healthy donors. The confusion matrix (*a*) summarizes the accuracy (positive predictive value (PPV) and false discovery rate (FDR)). The parallel coordinate plots (*b*) indicate the correlation between the four investigated biophysical cell properties (correct classified on top and incorrect classified below), showing an overall accuracy of 85.1%.

**Figure 6.**
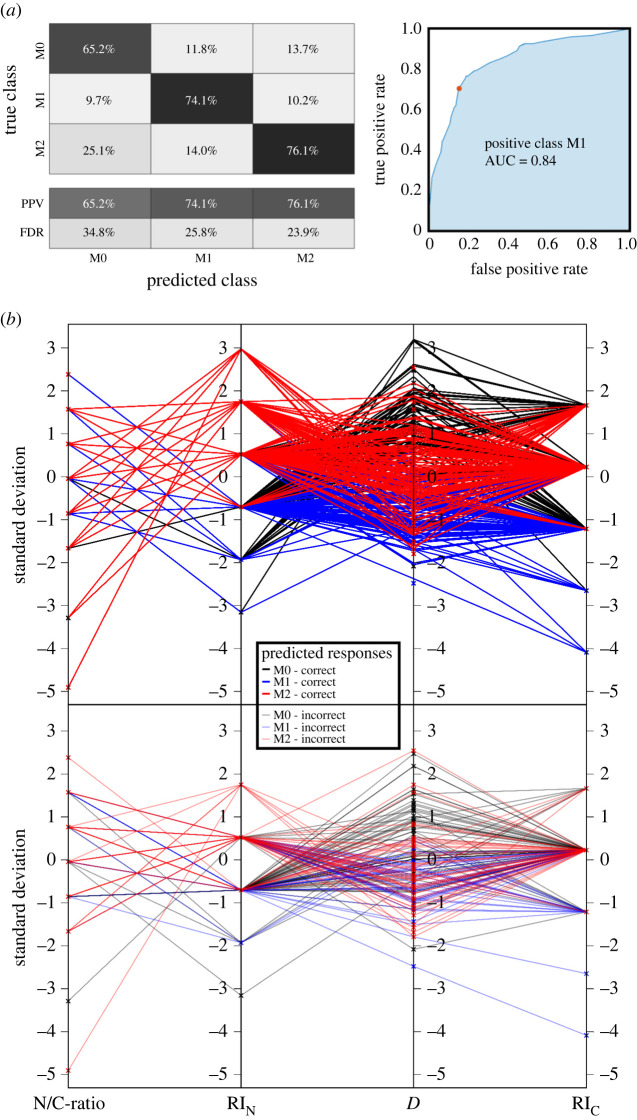
ML classification of M0 versus M1 versus M2 macrophage phenotypes from scattering outcome recovered from three healthy donors. The confusion matrix (*a*) summarizes the accuracy (positive predictive value (PPV) and false discovery rate (FDR)). The parallel coordinate plots (*b*) indicate the correlation between the four investigated biophysical cell properties (correct classified on top and incorrect classified below), showing an overall accuracy of 72.3%.

Alongside the investigation of optical signatures in three-dimensional scatter plots and the handmade classification, we developed a machine learning (ML) routine to predict an automated cell classification accuracy of our measurement apparatus to distinguish macrophages from monocytes and, moreover, (un-)polarized macrophage phenotypes (M0, M1 and M2) from each other (figures 5 and 6).

Monocytes (*n* = 107) [38] show significantly different optical cell signatures compared with M0 macrophage phenotype (*n* = 149) [38] as reported in the three-dimensional scatter plots (figure 4). As expected, a ML prediction accuracy greater than or equal to 99.2% was reached for e.g. linear, quadratic or cubic support vector machine (SVM) classifier using the following parameters: cross-validation = 5, misclassification cost = 1, box constraint level = 1, multi-class method = one-versus-one) without principal component analysis resulting in a prediction speed approximately 19 000 observation s^−1^ and training time approximately 0.24 s.

Next, we trained a ML algorithm with the biophysical cell properties of (un-)polarized macrophage cell subclasses. First, we trained M1 (*n* = 167) [38] versus M2 (*n* = 215) [38] macrophage phenotypes, where we found that the quadratic SVM classifier resulted as the most suitable algorithm according to calculation speed and prediction accuracy (training time approximately 0.51 s with approximately 31 000 observations s^−1^ and a total misclassification cost of 60) using the following ML parameters: one neighbour, Euclidean metric distance, equal distance weights, box constraint level of 1 and a kernel scale of 1. In fact, such ML parameters are used as a standard penalty for margin-violating observations, to prevent significant overfitting of the experimental data. We repeated the classification process five times resulting in an average prediction accuracy of approximately 85.1%. Other classifiers resulted in lower performances: the linear SVM-trained classifier showed an accuracy of approximately 84.3%, fine K nearest neighbour (KNN) approximately 78.3% and medium tree approximately 80.1%. The presented classifier shows a positive prediction for M1 cells of 83.2% and 85.1% for M2 cells (figure 5*a*). In addition, the parallel coordinate plots in figure 5*b* highlight the correlation between the four extracted biophysical cell properties, where each property is represented by a vertical axis.

Second, we trained M0 (*n* = 149) versus M1 (*n* = 167) versus M2 (*n* = 215) macrophage phenotypes, where we obtained that the cosine KNN classifier resulted as the most suitable algorithm according to calculation speed and prediction accuracy (training time approximately 0.25 s with approximately 25 000 observations s^−1^ and a total misclassification cost of 147) using the following ML parameters: 10 neighbours and equal distance weights. Also, in this case, we repeated the classification process five times, resulting in an average prediction accuracy of approximately 72.3%. Other classifiers resulted in lower prediction performances for the investigated macrophage phenotypes: the linear SVM-trained classifier showed an accuracy of approximately 71.7%, fine K nearest neighbour (KNN) approximately 64.2% and medium tree approximately 70.2%. The prediction accuracy results in a PPV of 65.2%, 74.2% and 76.1% for M0, M1 and M2, respectively (figure 6*a*).

Our results indicate that the mentioned ML classifier has better sensitivity in classifying M2 macrophage phenotype cells compared with other cells, due to the significantly different combination of biophysical properties (figure 6*b*). This could be ascribable to the different chromatin condensation and mitochondria presence in M2 cells, compared with other investigated cells.

## 3. Conclusion

Image-based machine learning is widely used in research and therapeutic applications, while the label-free investigation of scattering data is still underrated. We highlight in this work the potential of a simple and cost-effective microfluidic-based macrophage phenotype (unpolarized versus polarized) classification approach. Therefore, we analysed first in quiescent and afterwards in-flow condition the biophysical properties of polarized macrophage phenotypes as well as monocytes. Such living cell investigation resulted in a distinctive optical signature, which we used as input for a supervised ML-based cell classification. The analyses of more than 600 cells from three different donors allowed to predict macrophage phenotypes with an accuracy above 72% for M1 versus M2 macrophages and even more than 85% for M0 versus M1 and M2 phenotypes. Such outcome raises the hope for real-time-based cell analysis approaches based on scattering patterns. We believe that our measurement approach can be of significant therapeutic interest, where an identification, quantification and monitoring of both M1 to M2 phenotype is needed.

## 4. Material and methods

### 4.1. Cell collection

Human macrophages were recovered from healthy donors after obtaining informed consent, in accordance with relevant guidelines and regulations. In more detail, a standard venepuncture procedure was performed using standard K_2_EDTA tubes (Vacutainer, BD) to prevent coagulation. After sample collection, a standard density gradient separation was performed as followed: first, the whole volume of blood was diluted with an equal volume of phosphate buffered saline (PBS, Euroclone), and then gently layered on with an equal volume fraction of density gradient medium (Histopaque-1077, Sigma Aldrich) using a 50 ml centrifuge tube (Falcon). After that, a centrifugation step was performed at $300\vec{g}$ for 30 min and disabled machine brake. After the centrifugation, the resulting peripheral blood mononuclear cells (PBMC) were visible as a ring at the interface between the gradient medium (lower part) and the blood plasma (upper part). PBMC were collected with a disposable Pasteur pipette and washed in 10 ml of erythrocyte lysis buffer, to eliminate a possible contamination. Finally, cells were cultured in RPMI-1640 medium, supplemented with 10% fetal bovine serum, 1% L-Glu and 1% penicillin/streptomycin (Euroclone).

### 4.2. Macrophage phenotype polarization

PBMC were divided into three culture flasks (T-75, Corning) of equally distributed volume fractions to transform monocytes in unpolarized (M0), M1-polarized (M1) and M2-polarized (M2) macrophage phenotypes. After 24 h of incubation at 37°C and 5% CO_2_, cells in suspension (lymphocytes) were discarded, while adherent monocytes were treated for the following macrophage differentiation (day 0). First, cell medium was aspirated from the flask and substituted with RPMI-1640 and specific macrophage phenotype generation media (M0 = C-28057; M1 = C-28055; M2 = C-28056, Promocell). Complete cell medium was made of base medium with supplement mix and cytokines, following the manufacturer's instructions (Promocell). After 6 days (day 6) each flask was supplied with a volume of cell medium equal to 75% of the initial cell volume (day 0). At day 7 a new aliquot of cytokine mix (Promocell) was added to the medium. At day 9 the cell medium of each flask was aspirated to eliminate possible suspended cells, and a fresh volume of appropriate cell medium was added to each flask. At day 10, polarized and not-activated macrophages were detached from flask surfaces using a cell scraper tool and subsequently centrifuged at $200\vec{g}$ for 10 min in 15 ml centrifuge tubes (Falcon). Finally, cells were resuspended into 200 µl of complete RPMI-1640 medium, ready to be analysed with our optical cell investigation approach.

### 4.3. Microfluidic device and cell alignment

Cell measurements were performed with a microfluidic device, composed of a supporting geometry fabricated with a three-dimensional printer (Objet30 pro, Stratasys) and a series of two glass channels. Briefly, a round-shaped glass channel (TSP050375, Molex)—where three-dimensional alignment of cells takes place—is inserted on one side in a square shaped readout channel (8240, Vitrocom)—where single cell investigation takes place—which permits the precise in-flow optical readout of cells. The other end of the round-shaped channel is immersed in the cell sample. By applying a certain pressure on the sample liquid, the cell medium is pushed through the channel and enters the microfluidic device. Such sample liquid consists of cells immersed in an alignment medium, consisting of a highly biocompatible viscoelastic polymer (polyethylene oxide, PEO, molecular weight = 4 MDa, Sigma Aldrich) diluted in PBS at 0.4 wt%. Thanks to the resulting fluid properties, generated by viscoelastic fluid forces, cells are strictly aligned to the centreline of the round-shaped channel and subsequently remain aligned at the centreline of the subsequent microfluidic readout channel. Note that fluid forces have been chosen to prevent cell deformation effects, while ensuring sufficient single cell alignment. In more detail, three-dimensional cell alignment is achieved if the following relationship $3Wi\;\beta^{2}(L/2R)>-ln(3.5\beta )$ is satisfied. Where $Wi=2\lambda \bar{U}/2R$, uses λ the relaxation time (0.197 ms) of the viscoelastic fluid, the average fluid velocity (1496 µm^−1^), *R* the channel radius (25 µm), $\beta =r_{1}/R$, a non-dimensional geometrical channel parameter, with $r_{1}$ being the cell radius and *L* the channel length (0.35 m). However, the subsequent readout channel allows precise single cell analysis due to its square shape of 400 × 400 μm and preserved three-dimensional alignment. To ensure continuity between the alignment and readout channel, the alignment section is collinearly inserted in the readout section and sealed with a soft ferrule (UP-N-123–03X, Idex). At the end of the readout channel, cells can be recovered for further cell studies.

### 4.4. Sample preparation and observation

Cells are diluted in alignment medium to obtain a final cell concentration of approximately 1 × 10^5^ cells ml^−1^. Such cell concentration ensures a throughput rate of approximately 2 cells s^−1^ passing through the readout laser beam. Please note that the sample concentration and fluid velocities were optimized to reduce possible cell–cell interactions and cell deformation effects, while the maximum throughput performance of the actual measurement approach is approximately 50 cells s^−1^. Finally, each investigated macrophage phenotype is checked for mycoplasma infection. For off-chip cell investigations, macrophage types were observed with an inverted microscope (IX81, Olympus), to check their morphometric status before light scattering analysis. Therefore, a small volume of 10 µl was collected and observed using a 100× magnification and CMOS camera system (Orca flash 4.0, Hamamatsu Photonics). Since in suspension, cells were considered as healthy when they preserved their round shape without significant alterations at the external cell membrane. In addition, morphological cell alterations were investigated after immunostaining using a confocal microscope (TCS STED CW, Leica). For immunostaining, cells were first fixed with 4% paraformaldehyde (Sigma Aldrich) for 15 min at room temperature, then rinsed twice with PBS. Afterwards, permeabilization—with 0.1% Tryton X-100 (Sigma Aldrich) for 5 min—was performed and cell nucleus (Sytox Green, Thermo Fisher Scientific) as well as cell membrane (Cell Mask orange, Thermo Fisher Scientific) were investigated using µ-slide chambers (Ibidi).

### 4.5. Reverse transcription polymerase chain reaction analysis

To distinguish different macrophage phenotypes (M0-unpolarized, M1- and M2-polarized), reverse transcriptase polymerase chain reaction (RT-PCR) was carried out. To preserve the integrity and to stabilize the RNA for molecular analysis, freshly isolated monocytes derived from PBMCs (0.5·105 cells) were centrifuged at $12\;000\vec{g}$ for 2 min, the supernatant was removed, and the pellet was immediately suspended in 0.1 ml of RNAlater solution (Sigma Aldrich). The samples were stored at 4°C overnight (to allow the solution to thoroughly penetrate the tissue), then archived at −80°C. For RNA isolation, the RNAlater solution was diluted by adding an equal volume of ice-cold PBS to reduce the density of the solution avoiding damage to the cells and then centrifuged at normal speeds ($5000\vec{g}$). Then, total RNA was isolated using RNeasy plus mini kit (QIAGEN). Agarose gel electrophoresis was used to determine RNA integrity and RNA concentrations were examined by UV-light imaging system (Bio-Rad). Two hundred nanograms of total cellular RNA were reverse-transcribed (expand reverse transcriptase, Roche Diagnostics) into complementary DNA (cDNA) using random hexamer primers at 42°C for 45 min (random hexamers, Roche Diagnostics), according to the manufacturer's instructions. cDNA (2 µl) was amplified in a reaction mixture containing 10 mM Tris-HCl (pH 8.3), 1.5 mM MgCl_2_, 50 mM KCl, 200 µM dNTPs and 2.5 U of Taq DNA polymerase (Roche Diagnostics) in a final volume of 50 µl. The reaction was carried out in a DNA thermal cycler (Applied Biosystem). The expression of IL-10, CD68, and glyceraldehyde-3-phosphate dehydrogenase (GAPDH) used as a housekeeping gene was examined. Amplification primers were: IL-10, forward 5′-atgccccaagctgagaaccaagaccaa-3′ and reverse 5′-tctcaaggggctgggtcagctatccca-3′; CD68, forward 5′-gcaactcgagcatcattctttcacc-3′ and reverse 5′-gatgagaggcagcaagatgga-3′; GAPDH, 5′-ccacccatggcaaattccatggca-3′ and 5′-tctagactggcaggtcaggtccacc-3′. The amplification protocol consisted of an initial denaturation step at 95°C for 1 min. The cycles used for the primers were: 95°C for 1 min, 58°C for 1 min, for 35 cycles for IL-10; 95°C for 1 min, 51°C for 1 min, for 33 cycles for CD68. PCR products were analysed by electrophoresis on 1.8% agarose gel in TBE buffer 0.5×. Densitometric analysis of agarose gel stained with ethidium bromide was carried out measuring the band's intensity using ImageJ software and normalized by the housekeeping gene GAPDH. All samples were repeated three times.

### 4.6. Experimental set-up and image processing

We used a small-angle light scattering technique combined with a viscoelastic microfluidic single-cell alignment approach [30–32] In more detail, our cell investigation approach reveals biophysical properties of living cells from individual cell scattering records generated in a continuous angular range from approximately 2°−30° and an angular resolution of approximately 0.1°. Briefly, cells flowing in the readout channel of the microfluidic device pass through a collimated laser beam (*λ* = 632.8 nm). The resulting scattered light is collected and mapped on a camera sensor (ORCA Flash 4.0, Hamamatsu Photonics). The recorded scattering signatures are processed by a self-written Matlab (R2020b, MathWorks) routine to directly obtain the searched-for light-scattering profile (LSP) and consequently the biophysical cell properties of each passing cell. More specifically, collected LSPs are matched with a lookup table (greater than or equal to 335 000 curves) of previously calculated theoretical LSPs to obtain biophysical cell properties (diameter, *D*; Nucleus/cytoplasm-ratio, N/C-ratio; refractive index of the nucleus and cytoplasm, RI_N_ and RI_C_, respectively) and to distinguish morphological properties within the sub-micrometric cell dimension range. More detailed information about the LSP matching is shown elsewhere [30].

### 4.7. Machine learning

The machine learning (ML) approach was carried out with a Matlab (R2020b, MathWorks) routine to classify circulating monocytes from macrophages, as well as the main subtypes of macrophage phenotypes (M0, M1 and M2) based on their biophysical properties retrieved from optical cell signatures. Several classification methods were set up with operational parameters chosen based on previous experiments of our working group [31,33]. For ML training, a randomly chosen subset of data (all donors) was used, followed by testing classification accuracy on the remaining data (all donors), while the classification accuracy was measured by fivefold cross-validation. The ML results are shown in a 2 × 2 matrix for M1 versus M2 and 3 × 3 matrix for M0 versus M1 versus M2, with the following values: positive predictive value (PPV) and false discovery rate (FDR).

### 4.8. Statistical analysis

All results are presented as the mean ± standard error. When normality assumptions were met, the statistical significance for two or more groups of data was calculated by using a one-way ANOVA with corresponding Tukey's multiple comparison. Significance is indicated by *p* values (^ns^*p* > 0.05; *p < 0.05, **p < 0.01, ***p < 0.001) combined with *F*-values (*F*). We used Excel 365 (Microsoft Corporation) for all statistical analyses [34–36].

## Data Availability

Datasets for this research work are available from the Dryad Digital Repository: doi:10.5061/dryad.1ns1rn8wh [38].
